# Posterior cortical atrophy: visuomotor deficits in reaching and grasping

**DOI:** 10.3389/fnhum.2013.00294

**Published:** 2013-06-21

**Authors:** Benjamin P. Meek, Paul Shelton, Jonathan J. Marotta

**Affiliations:** ^1^Perception and Action Laboratory, Department of Psychology, University of ManitobaWinnipeg, MB, Canada; ^2^Department of Internal Medicine, Section of Neurology, University of ManitobaWinnipeg, MB, Canada

**Keywords:** posterior cortical atrophy, optic ataxia, grasping movements, Balint's syndrome, visuomotor deficits

## Abstract

Posterior Cortical Atrophy (PCA) is a rare clinical syndrome characterized by the predominance of higher-order visual disturbances such as optic ataxia, a characteristic of Balint's syndrome. Deficits result from progressive neurodegeneration of occipito-temporal and occipito-parietal cortices. The current study sought to explore the visuomotor functioning of four individuals with PCA by testing their ability to reach out and grasp real objects under various viewing conditions. Experiment 1 had participants reach out and grasp simple, rectangular blocks under visually- and memory-guided conditions. Experiment 2 explored participants' abilities to accurately reach for objects located in their visual periphery. This investigation revealed that PCA patients demonstrate many of the same deficits that have been previously reported in other individuals with optic ataxia, such as “magnetic misreaching”—a pathological reaching bias toward the point of visual fixation when grasping peripheral targets. Unlike many other individuals with optic ataxia, however, the patients in the current study also show symptoms indicative of damage to the more perceptual stream of visual processing, including abolished grip scaling during memory-guided grasping and deficits in face and object identification. These investigations are the first to perform a quantitative analysis of the visuomotor deficits exhibited by patients with PCA. Critically, this study helps characterize common symptoms of PCA, a vital first step for generating effective diagnostic criteria and therapeutic strategies for this understudied neurodegenerative disorder.

## Introduction

Posterior Cortical Atrophy (PCA) is a rare clinical syndrome characterized by prominent higher-order visual dysfunction, preserved memory and reasoning, and an insidious, often presenile onset (Zakzanis and Boulos, 2001; Mendez et al., 2002). The syndrome results from progressive cortical neurodegeneration that primarily targets occipital, parietal, and posterior temporal cortices. In the majority of cases, Alzheimer's pathology is the underlying cause, but cases have been documented as a result of corticobasal degeneration, dementia with Lewy bodies, and prion diseases such as Creutzfeldt-Jakob disease (Renner et al., 2004; McMonagle et al., 2006). Despite its close pathological connection to typical Alzheimer's disease (tAD), anatomical scans reveal a unique and distinguishable pattern of regional degeneration in individuals with PCA compared to those with tAD (Benson et al., 1988). Specifically, PCA patients show predominant areas of atrophy and hypometabolism extending from the primary visual cortex through the dorsal visual association cortex (Nestor et al., 2003; Whitwell et al., 2007). PCA also affects posterior regions of the temporal lobes, although this atrophy is comparable to that seen in cases of tAD (Whitwell et al., 2007). Due to PCA's progressive nature, patients will often experience more generalized cognitive losses later in the disease as atrophy spreads to anterior regions of the brain. Indeed, recent studies suggest that the clinical presentations of tAD and PCA may converge in later stages (Lehmann et al., 2012).

The most common symptoms of PCA include alexia, apperceptive visual agnosia, Balint's syndrome (simultanagnosia, optic ataxia, and ocular apraxia), Gerstmann's syndrome (agraphia, acalculia, left-right confusion, and finger agnosia), ideomotor apraxia, anomia, and visual field deficits. Less common symptoms, such as spontaneous Parkinsonian symptoms and visual hallucinations, may develop later in the disorder and may be indicative of a specific underlying pathology or diagnosis, such as dementia with Lewy bodies (Tang-Wai et al., 2003, 2004; McMonagle et al., 2006). As a predominant characteristic of Balint's syndrome, visuomotor deficits were an important feature of Benson et al.'s initial description of PCA in 1988, and disturbances in visuomotor functioning continue to be frequently reported as part of this disorder (e.g., Tang-Wai et al., 2003; Caine, 2004). The exact frequency of visuomotor disturbances in PCA is unknown, but some idea of its prevalence can be gleaned from previous group studies. For example, Mendez et al. (2002) reported the presence of optic ataxia in 11 of their 15 PCA patients, while Tang-Wai et al. (2004) observed optic ataxia in 10 of the 40 individuals they tested. The commonality of visuomotor dysfunction in cases of PCA is hardly surprising, however, when we consider that optic ataxia has been suggested as a defining symptom of the disorder and is often used as a key factor in diagnosing an individual with PCA.

Initially described by Balint in 1909, Balint's syndrome consists of simultanagnosia, optic ataxia, and ocular apraxia, which occur as a result of large legions in posterior parietal cortex (PPC). Patients with optic ataxia often demonstrate an inability to execute accurate visually-guided goal-directed reaching and grasping movements despite being free of impaired visual acuity or visual field deficits and having intact primary sensory and motor systems (Karnath and Perenin, 2005). Visuomotor deficits are apparent in both the proximal and distal components of grasping movements, including: prolonged movement times (Binkofski et al., 1998), inaccurate reaches to visual targets (Cavina-Pratesi et al., 2010), a disturbance or complete lack of in-flight hand shaping for object size (Jakobson et al., 1991), an inability to make fast corrective movements while reaching to perturbed stimuli (Grea et al., 2002), abolished implicit obstacle avoidance (Schindler et al., 2004), and inappropriate wrist-orientation for the task demands (Perenin and Vighetto, 1988). Optic ataxia can result from unilateral parietal damage to either hemisphere, with the resulting visuomotor problems primarily affecting the contralesional hand and the contralesional visual hemifield. Alternatively, optic ataxia can result from bilateral damage, with both hands and both hemifields being affected (Karnath and Perenin, 2005).

The areas of cortex surrounding the intraparietal sulcus (IPS) that are known to be damaged in cases of optic ataxia make up part of a neural network that is responsible for the visual guidance of our fast and accurate interactions with the objects around us. The two-stream theory of visual processing labels this pathway as the dorsal stream, since visual information projects dorsally from primary visual cortex to parietal association cortex (Ungerleider and Mishkin, 1982; Goodale and Milner, 1992). This dorsal pathway allows for the conversion of incoming visuo-spatial information into a body-centric coordinate system, as well as for the planning and coordination of object-interactive movements. In contrast, the ventral stream of visual processing, which projects from the occipital lobe into posterior temporal cortex, is responsible for the identification and recognition of objects. Specialized areas in this pathway, such as inferior temporal (IT) cortex, allow for the integration of visual information into a vivid and robust representation of an object. The ventral stream also prepares these object “percepts” for long-term storage and later retrieval from memory. Although the dorsal and ventral streams are functionally and architectonically distinct, they are highly interconnected and share large amounts of information (e.g., Ramayya et al., 2010).

Milner et al. (2003) demonstrated that a select few optic ataxic patients, who cannot properly scale their grip during visually guided movements, show a paradoxical improvement in their performance when vision is removed and a delay is introduced between object viewing and movement execution. A number of groups have shown that introducing a delay into a pointing task produces a similar improvement in pointing accuracy for optic ataxic patients (Milner et al., 1999a, 2001; Revol et al., 2003; Himmelbach and Karnath, 2005). It has been suggested that this change in behavior results from a shift in the neural control of the action from real time visuomotor control systems in the dorsal stream to stored perceptual object representations in the ventral stream (Milner et al., 2003). This theory has been supported by complementary evidence from experiments involving DF, a patient with profound perceptual deficits due to bilateral damage to her ventral stream (Milner et al., 1991; Murphy et al., 1998). Even though DF can perform a real time grasping task without problem (Goodale et al., 1991, 1994), her ability to properly scale her grip when performing a grasp following a delay is almost completely nonexistent (Goodale et al., 1994). Similarly, DF produces much larger errors than controls when pointing after a delay compared to her excellent performance on the same task in real time (Milner et al., 1999b). It should be noted that DF also exhibits considerable atrophy in some areas of her parietal lobes, including regions of her left-hemisphere thought to control reaching and grasping functions (James et al., 2003). James et al. (2003) suggest that DF's relatively preserved visuomotor abilities may be a result of a reorganization of reach- and grasp-related functions to the right hemisphere, as suggested by significant ipsilateral activation during right handed reaching.

Even with bilateral lesions, individuals with optic ataxia usually exhibit reaching and grasping deficits only when performing tasks in their peripheral visual fields. In other words, when allowed to orient their eyes toward a target, patients' visuomotor performances are often no different from those of controls (Himmelbach et al., 2006). Although this pattern of behavior is the most common consequence of optic ataxia (Buxbaum and Coslett, 1997), it is not always the case; patients with optic ataxia have been known to demonstrate deficits in reaching and grasping under foveal guidance (Perenin and Vighetto, 1988; Binkofski et al., 1998). Additionally, it has been documented that patients who show reaching mislocalizations due to damage to posterior parietal brain areas—both from acute injuries as well as degenerative disorders—often demonstrate a reaching bias toward the point of fixation when confronted with targets located in their visual peripheries (Ratcliff and Davies-Jones, 1972; Carey et al., 1997; Jackson et al., 2005). Meanwhile, there is evidence that normal controls show the opposite behavior—a horizontal “overshoot” away from the point of fixation (Henriques et al., 1998; Henriques and Crawford, 2000; Khan et al., 2004).

Although many studies have explored the prevalence and presentation of the key symptoms of PCA, a quantitative investigation into the visuomotor functioning of individuals with the disorder has yet to be performed. Instead, the presence of optic ataxia in cases of PCA has predominantly been identified based on qualitative observations of misreaching (e.g., Goethals and Santens, 2001; Mendez et al., 2002). The main goal of this study was to perform a detailed quantitative analysis of the grasping abilities of a small group of patients with PCA. Specifically, we sought to determine whether the visuomotor deficits that arise because of PCA are similar to those previously documented as a result of optic ataxia due to alternate pathological processes. Experiment 1 was designed to first measure the kinematics of reaches made by individuals with PCA to simple, symmetrical objects at midline under free-viewing conditions. Subsequently, Experiment 1 sought to test whether these same individuals show any reduction or improvement in grasping performance following the introduction of a delay between object presentation and a reach-to-grasp movement. Given the wide-ranging perceptual problems in PCA, we hypothesized that our PCA patients' reaching and grasping performance would not improve following a delay. Experiment 2 sought to investigate whether individuals with PCA show similar visuomotor impairments to other patients with optic ataxia when reaching for objects located in their peripheral visual fields. Based on previous qualitative reports of peripheral pointing errors in individuals with PCA, we expected to see peripheral reaching and grasping errors. However, the exact manifestations of these errors were of interest, as were the quantitative analyses of reach trajectories to objects at midline.

## Materials and methods

### Statement on ethics

Procedures were reviewed and approved by the Human Research Ethics Board at the University of Manitoba.

### Participants

#### Patient information

**Figure 1:**
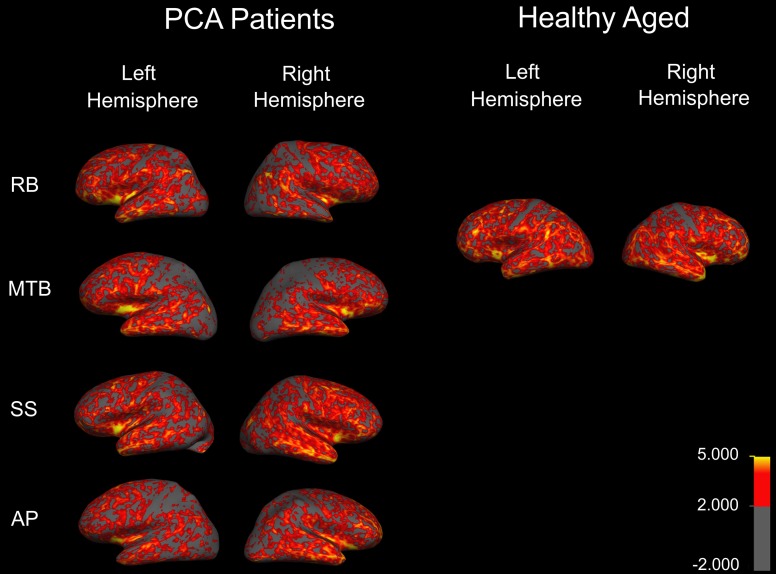
**Cortical thickness map obtained in the four patients with PCA**. Maps are overlaid on inflated brains, so as to display thickness of cortex in sulci. A color spectrum was applied with yellow (5 mm) and red (greater than 2 mm) indicating areas of thicker cortex and gray indicating thin cortex (less than 2 mm).

Four individuals with PCA were recruited for the current study. All four individuals received their diagnosis from a local neurologist (P.S.) based on cognitive and perceptual testing combined with structural imaging data (Figure 1). All participants had normal or corrected-to-normal visual acuity, as determined by either their neuro-opthalmologist or optometrist. Participants underwent an initial battery of basic visual, motor, and cognitive tests. A basic Edinburg handedness inventory (Oldfield, 1971) was run to determine hand-dominance. Visual perception was evaluated using a number of established tests: Benton face task (Oxford University Press, New York, NY), Benton Visual Form Discrimination task (VFDT; Oxford University Press, New York, NY), Benton Line Orientation task (Oxford University Press, New York, NY), and the Boston naming task (Pro-ed, Austin, TX). Custom made tasks for “object counting” and “object naming” were also run. Together, these tasks provided a reasonable account of participants' perceptual abilities—from basic shape discrimination to more complex face perception.

Two tests of reaction time were administered as measures of psychomotor processing speed (Salthouse, 2000): a repetitive finger tapping task (Veeder-Root, Hartford, CT) and a simple reaction time task. The reaction time task was custom made and programmed in E-Prime software (Psychology Software Tools, Pittsburgh, PA). Basic cognitive abilities were assessed using the Mini Mental State Examination (MMSE; Folstein et al., 1975) and the Dementia Rating Scale (DRS-2; Psychological Assessment Resources, Lutz, FL). A summary of the results of these tests can be found in Table 1.

**Table 1:** **Demographic information and behavioral scores of four patients with PCA**.

	**RB**	**MTB**	**SS**	**AP**
Age	76	67	66	78
Sex	F	F	M	F
Handedness	Right	Right	Right	Left
MMSE	26 (normal)	30 (normal)	30 (normal)	28 (normal)
DRS-2	126, 6–10th percentile (mildly impaired)	125, 3–5th percentile (moderately impaired)	129, 6–10th percentile (mildly impaired)	133, 29–40th percentile (below average)
Reaction time (Median)	560 ms	672 ms	717 ms	642 ms
Finger tapping (Right)	19 taps/10 s	15.3 taps/10 s	21.5 taps/10 s	5.8 taps/10 s
Finger tapping (Left)	18.5 taps/10 s	11.83 taps/10 s	21.2 taps/10 s	14.3 taps/10 s
Object counting	12/14	3/14	6/14	11/14
Object identification	5/18	18/18	8/18	18/18
Boston naming	1/15	3/15	7/15	14/15
Benton line orientation	0/5	Unable to perform	2/30	7/15
Benton VFDT	1/32	Unable to perform	5/14	19/32
Benton FRT	38/54 (moderately impaired)	29/54 (severely impaired)	34/54 (severely impaired)	45/54 (normal)

Abbreviations: MMSE, Mini-Mental State Examination; DRS-2, Dementia Rating Scale; Benton VFDT, Benton Visual Form Discrimination Task; Benton FRT, Benton Facial Recognition Test.

To examine the extent and distribution of atrophy in all of the PCA patients, MRI scans were performed and analysed for each patient individually. Thickness maps were rendered onto an inflated brain template to reveal measurements of cortex in the sulci (Figure 1). A color spectrum was applied with yellow indicating areas of thicker cortex (5 mm) and gray indicating thin cortex (less than 2 mm). For additional methodological details, please see accompanying paper (Meek et al., 2013).

***Patient RB.*** RB is a 76 year-old female who first demonstrated symptoms of PCA three to four years prior to testing. These difficulties initially manifested as trouble recognizing recent photographs of family members and friends, but soon progressed to visual color disturbances and more severe issues with face and object recognition. RB received a diagnosis of probable PCA based on the following clinical observations: intact memory and executive functioning (as determined by MMSE and DRS-2), prosopagnosia, visual object agnosia, simultanagnosia, mild hemispatial neglect, achromatopsia, constructional apraxia, alexia, agraphia, and elements of Gerstmann's syndrome. Additionally, SPECT scans revealed moderate hypoperfusion in the right parietal lobe, extending to the posterior temporal lobe, with milder changes in the left parietal area. Hypoperfusion was also present in the IT cortex, extending to the angular gyrus on the right side. MRI scans revealed very mild diffuse cerebral atrophy. RB shows evidence of bilateral upper quadrantanopia, which is more pronounced in the right visual field.

***Patient MTB.*** MTB is a 67 year-old female who started to develop problems with her vision and motor coordination almost 8 years prior to testing. Since then, her visual and motor problems have progressed to the point where she no longer reads for pleasure or operates a vehicle. She has difficulty discriminating right from left, and has developed an unsteady gait. MTB is still able to read using a restricted set of stimuli. However, the process of reading is an arduous one, and she claims that the letters “jump around” on her. This same problem impairs her performance on basic copying tasks, as she complains that the object she is trying to draw is “dancing” on the page. MTB has also reported having trouble accurately interacting with objects when trying to pick them up. MTB received a diagnosis of probable PCA given her intact memory and reasoning capabilities in conjunction with simultanagnosia, mild hemispatial neglect, ideomotor apraxia, and constructional apraxia. SPECT scans revealed moderate bilateral parietal-occipital hypoperfusion, which is more pronounced in the right hemisphere, and MRI scans showed marked bilateral parietal atrophy. Visual field tests indicate the presence of left homonymous hemianopia, along with a generalized loss of sensitivity in the right visual field.

***Patient SS.*** SS is a 66 year-old male who first showed problems with basic navigation in 2007. Since then, he has gradually and progressively developed pronounced deficits in route finding and spatial orientation. SS sometimes has difficulty finding objects in front of him that seem to be in plain view. Despite being a retired accountant, SS can no longer perform simple calculations. SS demonstrates difficulties with reading and spelling, and he shows minor word finding and semantic paraphasic errors—sometimes replacing words with similar, but incorrect, words. SS shows some difficulty on tests of executive functioning—performing slowly at number cancellation, for example—but is not impaired on tests of executive function that are free of spatial elements. SS was given a diagnosis of probable PCA based on the following clinical observations: simultanagnosia, optic ataxia, hemispatial neglect, elements of Gerstmann's syndrome, ideomotor apraxia, constructional apraxia, visual object agnosia, prosopagnosia, alexia, agraphia, anomia, and aphasia. MRI scans revealed mild diffuse atrophy, while SPECT scans demonstrated severe bilateral inferior parietal lobe hypoperfusion. SS also shows some evidence of visual field defects, which are more apparent in the upper visual fields.

***Patient AP.*** AP is a 78 year-old, left-handed female who first visited us in 2009. A retired journalist, AP suffers from a variety of symptoms consistent with PCA. Her first and major complaint is difficulty reading; she reports that the words “jump” on her, and she struggles to find her place in a line of text. As is common in PCA, AP reports dressing apraxia—needing help to get her clothes turned around the right way before she can get them on. She also shows a mild increase in muscle tone and demonstrates dysarthria—sometimes struggling to generate fluent speech. AP's neurologist has suggested a diagnosis of probable PCA based on the following observations: intact memory and executive functioning, simultanagnosia, optic ataxia, bilateral hemispatial neglect, ideomotor apraxia, and constructional apraxia. AP also demonstrates slowed limb movements and right-side tactile extinction. MRI scans revealed moderate diffuse cortical atrophy, while SPECT scans showed moderate parietal-occipital hypoperfusion in the left hemisphere, along with marginal right parietal hypoperfusion.

#### Healthy age-matched controls

Eight age-matched, healthy individuals (seven females, one male; age range = 63–80 years old; mean age = 71.8 years old) were recruited to act as control subjects. All eight individuals participated in Experiment 1, with six of these same subjects participating in Experiment 2. The control group was also used to provide a “normal” range of results for two preliminary tests: reaction time and repetitive finger tapping. Controls produced an averaged median reaction time of 294 ms [95% confidence interval (CI): 200–388 ms]. On the repetitive finger tapping task, controls produced an average of 35.2 taps/10 s (95% CI: 16.6–53.8 taps/10 s) with their right hands, and 31.0 taps/10 s (95% CI: 16.3–45.6 taps/10 s) with their left hands.

### Methodology

#### Experiment 1—delayed grasping

Experiment 1 consisted of three conditions: closed-loop grasping, immediate open-loop grasping, and delayed open-loop grasping. In the closed-loop condition, participants were asked to reach out and pick up a single object with visual feedback of their actions fully available. In immediate open-loop grasping, participants were allowed to view the target object for three seconds before being given a cue-to-grasp as their vision was occluded. In delayed open-loop grasping, participants were first allowed to view the target object for 3 s before their vision was occluded. Once their vision was obscured, they were required to wait 3 s before the auditory cue-to-grasp was presented. Trials were blocked by condition, and the order in which conditions were performed was counterbalanced across participants.

In each of the three viewing conditions, symmetrical “Efron blocks” were placed on a tabletop in front of participants, at their midline. Participants were provided with a “start button”—a raised landmark 7 cm from the edge of the table—to which they were instructed to return their hand after each grasp. Between all trials, participants were told to keep their index finger and thumb together and resting on the start button, with their remaining fingers tucked comfortably against their palm. An auditory cue-to-grasp, a brief tone, indicated the start of each trial, at which point participants were free to initiate a grasp movement. Participants were required to execute a precision grasp to each object, using only their index finger and thumb in opposition, grasping the blocks across their vertical axis.

Efron blocks are small, rectangular, wooden objects with the same overall surface area but different geometric dimensions. Five different Efron blocks were used for this study: block A (length: 5.0 cm, width: 5.0 cm, height: 1.1 cm), block B (5.3, 4.5, 1.1 cm), block C (6.3, 4.0, 1.1 cm), block D (7.2, 3.5, 1.1 cm), and block E (8.0, 3.0, 1.1 cm). Only the data from blocks A, C, and E were analysed, while blocks B and D served as distracters to prevent participants from “ball-parking” the size of the three target objects. Blocks were placed on the tabletop directly in front of participants at one of three distances: 20, 30, or 40 cm from the edge of the table. Each of the three target blocks was presented 15 times in each condition—five times at each of the three distances, while the two distracter blocks were presented six times each—twice at each of the three distances. Participants made 57 grasps for each of the three conditions, for a total of 171 trials per testing session.

Position and velocity recordings were made using a portable Motion Monitor system (Innovative Sports Technology; Chicago, IL) attached to miniBirds magnetic sensors (Ascension Technology Company; Burlington, VT). Individual sensors were attached to the index finger, thumb, and wrist of each participant's dominant hand. Participants also wore liquid-crystal “shutter goggles” (PLATO Translucent Technologies; Toronto, Canada) over their regular eye-wear (if any), which could be remotely toggled between a transparent and an opaque state. Custom-written software, run off the Motion Monitor system, allowed for the timing of visual occlusion to be precisely controlled. The presentation of the auditory tone, used as the cue-to-grasp, was controlled by the same software responsible for switching the shutter goggles. As a result, the timing of the grasp-cue and visual occlusion were tightly coordinated in all conditions.

#### Experiment 2—peripheral grasping

Experiment 2 employed a very similar set-up to Experiment 1. Participants were seated directly in front of a dark-colored table and asked to reach out and pick up objects presented to them on the tabletop. For this task, participants were required to maintain fixation for the entirety of each trial at a central fixation point located on the tabletop at their midline. The fixation point was positioned 30 cm from the edge of the table. An experimenter seated opposite the subject ensured that fixations were maintained at the central fixation spot. If participants did not maintain fixation with the central point for the entirety of the trial, then that data was not used and the trial was repeated at a later time. Each trial consisted of one of the three target-blocks used in Experiment 1—block A, block C, and block E—being presented, one at-a-time, at one of three possible locations: the fixation point itself, 12 cm to the left of the fixation point, and 12 cm to the right of the fixation point. These peripheral grasp sites roughly corresponded to a 22° viewing-angle from the subject's location. When objects were presented at the two peripheral locations, their more proximal edges were positioned at the 12 cm distance, thereby ensuring that no part of any object fell closer than 12 cm to the subject's point-of-fixation, regardless of their size. Each of the three target-blocks was presented five times at each location, and the entire procedure was repeated for each hand. Thus, participants made 45 grasps with each hand, for a total of 90 trials per testing session. As in Experiment 1, position recordings were made using the portable Motion Monitor system attached to miniBirds magnetic sensors, with individual sensors affixed to the index finger, thumb, and wrist.

### Data analysis

Statistical tests adopted a type-1 error rate of α = 0.05, with calculated *p*-values being considered significant if they fell below this value. All confidence intervals were generated using statistics developed by Crawford and Garthwaite (2002), Crawford et al. (2010) for use when comparing single-subjects with small control groups.

#### Experiment 1—delayed grasping

Custom-written algorithms in Motion Monitor outputted the value of MGA for each grasp. A separate regression analysis was run for each participant under each condition (closed-loop, immediate, and delayed). This analysis was performed in order to reveal whether participants were scaling their grip apertures in relation to the size of the block, as shown by a regression slope significantly different from zero. In addition to MGA, peak velocity and movement duration were also recorded. The start of each trial was denoted by the time at which forward wrist velocity exceeded 0.05 m/s, and the trial ended upon object contact. Object contact was defined as the moment at which the thumb and index finger were both touching the object, as determined by the experimenter. A 95% confidence interval was generated surrounding the controls' mean value for each condition. Individual patient data was then plotted against these confidence intervals to determine if the patients' kinematics were different from controls. Additionally, single-subject ANOVAs were run for each patient to test for significant differences across conditions for each variable. *Post-hoc* analysis using Tukey's Honestly Significant Difference (Tukey's HSD) test determined the exact “location” of any main effects. Finally, each grasp was defined qualitatively in order to give a measure of how successful participants were at accurately guiding their hand and fingers to the target block in each condition. This accuracy was measured by categorizing each grasp as either “successful”—the hand was directed to the correct target location and the grasp was executed without corrections having to be made; “missed grasp”—the hand was directed to the correct target location, but corrections were required to acquire a stable grasp; and “missed block”—the hand was not directed to the correct target location.

#### Experiment 2—peripheral grasping

Custom-written analysis software was assembled using Python (Python Software Foundation, Beaverton, OR) in order to study the path of the hand as it moved from the start position to the target. All positional information was taken from the magnetic sensor attached to each participant's wrist. For each trial, an idealized, straight-line path was calculated between the start and end positions of the wrist. The deviation of the actual reach from this idealized path was then calculated by measuring the distance between the two paths at 30 equally-spaced points along the idealized line. This method enabled us to find the average path taken by a particular participant, or group of participants, within a particular condition by averaging the distance between the actual and idealized paths at each of the 30 points across multiple trials. By combining the behavior of all control participants, we were able to generate the “normal” path taken by controls to targets located at each position. The main variables of interest were the maximum leftward and rightward deviation of the reach from the idealized path for each trial. Ninety-five percent CI were generated for control data for each block location. These intervals allowed us to see whether reaches made by controls differed from the idealized, straight-line paths, as well as whether the reaches made by each patient differed significantly from the paths taken by controls.

To investigate the prominence of magnetic misreaching in movements directed toward peripheral targets, we recorded the number of trials in which the index finger passed through a 3 cm-radius region of interest (ROI) around the fixation point. The size of the ROI was determined prior to analysis based on the dimensions of the experimental setup. Three centimeters was deemed to be large enough to catch any reaches directed specifically toward the fixation point, but small enough to avoid counting “normal” reaches.

## Results

### Experiment 1—delayed grasping

#### Controls

**Figure 2:**
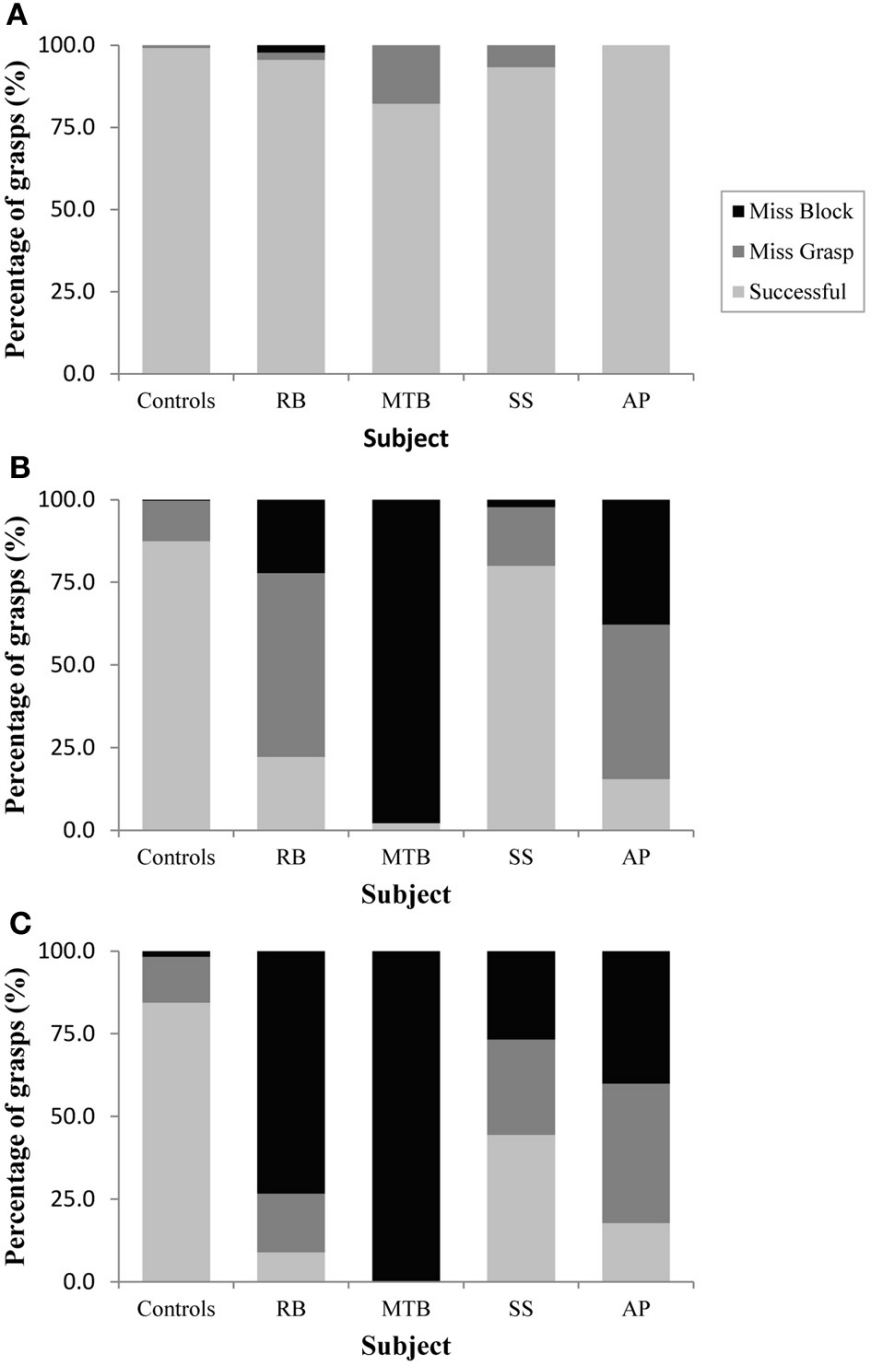
**Grasping precision in Experiment 1 for (A): the closed-loop condition, (B): the immediate open-loop condition, and (C): the delayed open-loop condition**.

The age-matched controls were able to perform a fluid, uncorrected grasp in 99.2% of trials in the closed-loop (free-viewing) condition (Figure 2A), and all control participants demonstrated grip scaling behavior—their MGAs decreased as the vertical size of the target blocks decreased (Table 2A). Removing visual feedback of the grasp lead to a slight increase in the number of grip corrections required to pick up the target blocks, but overall accuracy remained high in both the immediate open-loop and delayed open-loop conditions (see Figures 2B,C). When errors did occur, it was most common for mistakes to consist of minor grip adjustments following object contact; complete misses were rare. In the open-loop conditions—during which visual feedback of the reach was not available—controls showed a generally preserved ability to scale their grasps to the size of the target block; all controls showed significant scaling in the immediate open-loop condition (Table 2B), and only one control subject failed to scale in the delayed condition (Table 2C). However, the slope-values and coefficients of determination of the regression lines indicate that this scaling behavior was not as robust as during closed-loop trials.

**Table 2A:** **Regression values for Closed-loop grasping**.

	**Slope**	***R*^2^**	***p*-value**
RB	−3.004	0.048	0.152
MTB	−9.2	0.444	<0.001
SS	−5.2	0.232	0.001
AP	−3.633	0.295	<0.001
Control I	−5.467	0.634	<0.001
Control II	−4.533	0.395	<0.001
Control III	−9.467	0.599	<0.001
Control IV	−7.267	0.728	<0.001
Control V	−9.933	0.622	<0.001
Control VI	−8.767	0.846	<0.001
Control VII	−6.642	0.507	<0.001
Control VIII	−5.583	0.477	<0.001

**Table 2B:** **Regression values for Immediate Open-loop grasping**.

	**Slope**	***R*^2^**	***p*-value**
RB	1.532	0.032	0.243
MTB	−0.500	0.002	0.768
SS	−7.040	0.260	<0.001
AP	−3.067	0.128	0.016
Control I	−4.467	0.452	<0.001
Control II	−3.167	0.125	0.017
Control III	−4.467	0.263	<0.001
Control IV	−4.200	0.207	0.002
Control V	−5.967	0.232	0.001
Control VI	−10.267	0.724	<0.001
Control VII	−5.300	0.360	<0.001
Control VIII	−4.233	0.165	0.006

**Table 2C:** **Regression values for Delayed Open-loop grasping**.

	**Slope**	***R*^2^**	***p*-value**
RB	2.321	0.090	0.050
MTB	−1.500	0.027	0.313
SS	−2.733	0.048	0.146
AP	1.100	0.019	0.373
Control I	−5.833	0.477	<0.001
Control II	−1.600	0.105	0.030
Control III	−5.367	0.292	<0.001
Control IV	−2.633	0.115	0.023
Control V	−5.900	0.352	<0.001
Control VI	−6.667	0.746	<0.001
Control VII	−1.300	0.025	0.304
Control VIII	−3.800	0.371	<0.001

**Figure 3:**
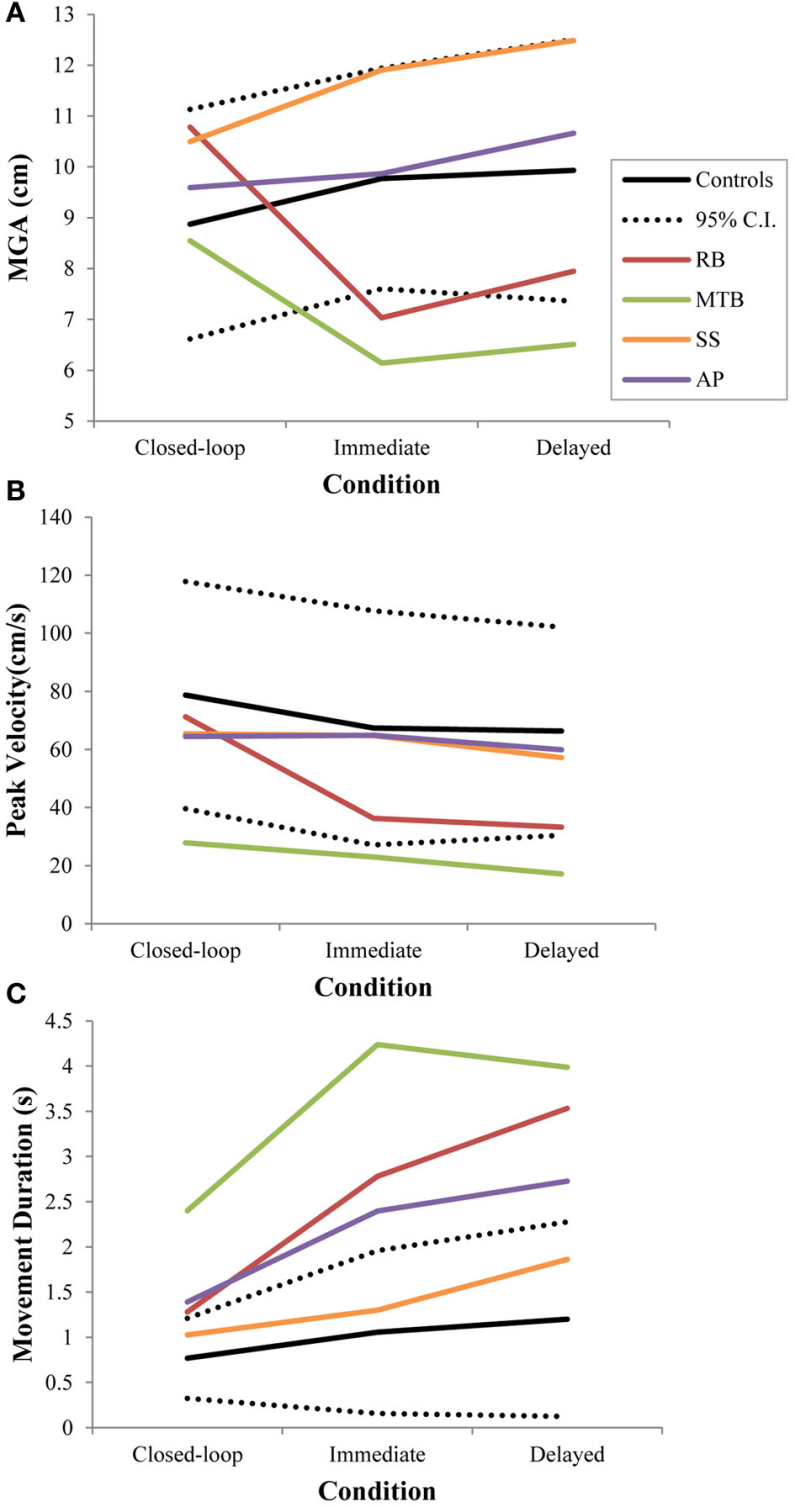
**Three different kinematic measures—(A): maximum grip aperture (MGA), (B): peak wrist velocity, and (C): movement duration—for patients and controls across all three conditions in Experiment 1**. The dotted lines represent the upper and lower limits of the 95% confidence interval for control data.

Controls did not show a significant change in overall MGA across conditions [*F*_(2, 21)_ = 2.673, *p* = 0.092], although there was a definite trend toward larger MGA values in the two open-loop conditions compared to closed-loop grasping (Figure 3A). There was also no significant change in peak velocity across conditions [*F*_(2, 21)_ = 3.013, *p* = 0.071; Figure 3B]. Again, however, there seemed to be a trend toward a change in behavior between the closed-loop and open-loop conditions, with controls showing a tendency to produce lower peak velocities during open-loop grasping (Figure 3B). The overall duration of reaches produced by controls did change significantly across conditions [*F*_(2, 21)_ = 3.825, *p* = 0.038; Figure 3C], likely due to the trend toward lower reach velocities in the open-loop conditions. *Post-hoc* analysis revealed that this main effect of condition was driven by a difference in reach duration between the closed-loop and delayed open-loop condition (*p* = 0.033).

#### Patients

***Patient RB.*** RB showed no evidence of scaling for the closed-loop [*r*^2^ = 0.048; *F*_(1, 43)_ = 2.134, *p* = 0.152] or immediate open-loop conditions [*r*^2^ = 0.032; *F*_(1, 43)_ = 1.405, *p* = 0.243]. Curiously, RB did demonstrate significant scaling in the delayed open-loop condition [*r*^2^ = 0.090; *F*_(1, 42)_ = 4.076, *p* = 0.050], but the scaling was in the opposite direction to what would be expected. In other words, her maximum grip aperture increased as the vertical size of the block decreased. RB's overall MGA dropped significantly from the closed-loop condition to the immediate and delayed conditions [*F*_(2, 131)_ = 34.193; *p* < 0.001]. *Post-hoc* analysis revealed that these main effects of condition were driven by significant differences in overall MGA between the closed-loop condition and both open-loop conditions, rather than between the two open-loop conditions (Figure 3A). However, it should be noted that RB's overall MGA did increase significantly between the immediate and delayed conditions (*p* < 0.001). RB also showed a large decrease in wrist velocity between the closed and open-loop conditions [*F*_(2, 133)_ = 89.651, *p* < 0.001]. Despite this large decrease in peak velocity for the open-loop conditions, RB's speeds fell within the 95% CI of control data (Figure 3B). RB produced much longer overall movement durations than controls for all conditions (Figure 3C), and also showed longer average movement durations in the open-loop conditions compared to closed-loop grasping [RB: *F*_(2, 134)_ = 46.675, *p* < 0.001]. Despite RB's failure to scale her grasps in the closed-loop condition, she was able to guide her fingers to appropriate grasp sites without correction on 95.6% of trials (Figure 2). However, this performance deteriorated greatly in the open-loop conditions, such that RB missed the target object completely on 73.3% of trials in the delayed open-loop condition.

***Patient MTB.*** In the closed-loop condition, MTB showed significant scaling [*r*^2^ = 0.444; *F*_(34, 379)_, *p* < 0.001], but this same scaling behavior was not present in the immediate or delayed open-loop conditions [immediate: *r*^2^ = 0.002; *F*_(1, 40)_ = 0.088, *p* = 0.768; delayed: *r*^2^ = 0.027; *F*_(1, 39)_ = 1.045, *p* = 0.313]. MTB's overall MGA dropped from 8.55 to 6.14 cm and 6.51 cm across the respective conditions [*F*_(2, 125)_ = 79.937; *p* < 0.001; Figure 3A]. As with RB, *post-hoc* analysis revealed that this main effect of condition was driven by significant differences in overall MGA between the closed-loop condition and both open-loop conditions, rather than between the two open-loop conditions. MTB showed significantly lower peak velocities than controls across all three conditions (Figure 3B). Despite her slower velocities, MTB still showed the same pattern of decreasing peak velocity across conditions [*F*_(2, 134)_ = 24.812, *p* < 0.001]. As might be expected from her slowed wrist velocity, MTB produced much longer overall movement durations than controls for all conditions (Figure 3C), and showed longer movement durations in the open-loop conditions compared to closed-loop grasping [*F*_(2, 134)_ = 17.573, *p* < 0.001]. Despite showing appropriate scaling in the closed-loop condition, MTB made slight grip corrections on the block for 17.8% of grasps (Figure 2). Additionally, she proved almost completely unable to locate objects without constant visual feedback of the scene, as she missed the target location on 98.9% of her grasps across both open-loop conditions.

***Patient SS.*** SS showed significant scaling behavior in both the closed-loop [*r*^2^ = 0.232; *F*_(1, 43)_ = 12.986, *p* = 0.001], and immediate open-loop conditions [*r*^2^ = 0.260; *F*_(1, 43)_ = 14.734, *p* < 0.001]. However, when a delay was introduced between object viewing and movement onset, SS's grasp apertures across blocks produced a regression line that was no different from zero [*r*^2^ = 0.048; *F*_(1, 44)_ = 2.190, *p* = 0.146]. The change in SS's MGA values across conditions resembled controls in that they increased steadily across conditions from closed-loop to immediate to delayed [*F*_(2, 133)_ = 44.449; *p* < 0.001; Figure 3A]. SS's peak velocity values fell well within the 95% CI of control data for all conditions (Figure 3B). Like controls, SS's peak velocity decreased across conditions from closed-loop grasping through to the delayed condition [*F*_(2, 134)_ = 3.441, *p* = 0.035]. SS was the only patient whose overall movement durations were comparable to controls (Figure 3C), and, like controls, SS showed a pattern of increasing durations across conditions [*F*_(2, 134)_ = 16.085, *p* < 0.001]. SS made corrections on relatively few of his grasps in the closed-loop and immediate open-loop conditions (Figure 2), but his delayed performance was poor, as he made minor grip corrections on 28.9% of his grasps and missed the block completely on 26.7%.

***Patient AP.*** AP showed scaling behavior in both the closed-loop [*r*^2^ = 0.295; *F*_(1, 43)_ = 18.022, *p* < 0.001] and immediate open-loop conditions [*r*^2^ = 0.128; *F*_(1, 44)_ = 6.292, *p* = 0.016]. However, like SS, this same behavior was not present when a delay was introduced between object viewing and movement onset [*r*^2^ = 0.019; *F*_(1, 44)_ = 0.811, *p* = 0.373]. AP's MGA values increased steadily from the closed-loop condition, to the immediate and delayed conditions [*F*_(2, 134)_ = 33.872; *p* < 0.001; Figure 3A], and her wrist velocities were comparable to controls, falling within the 95% CI of control data for all conditions (Figure 3B). Although AP showed very similar peak velocities to SS across conditions, there were no significant differences in these values [*F*_(2, 134)_ = 2.353, *p* = 0.099]. AP's overall movement durations were much longer than controls in all conditions (Figure 3C), and she showed longer movement durations in the open-loop conditions compared to closed-loop grasping [*F*_(2, 134)_ = 32.040, *p* < 0.001]. AP executed accurate grasps on all closed-loop trials, but required minor corrections of her grasps in the immediate and delayed conditions (Figure 2). Furthermore, AP missed the block completely on 37.8 and 40.0% of trials during immediate and delayed grasping, respectively.

### Experiment 2—peripheral grasping

#### Controls

**Figure 4:**
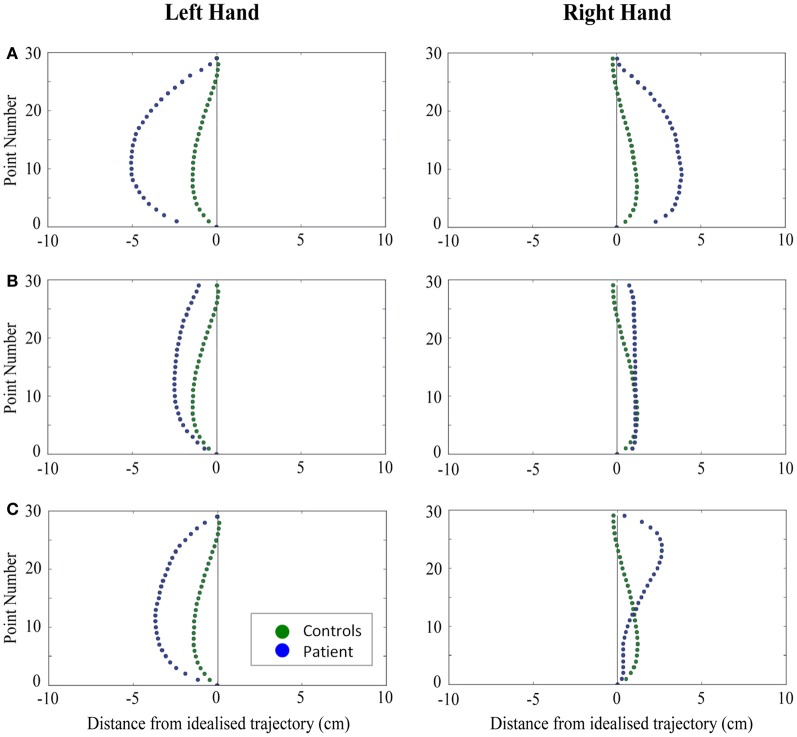
**A comparison of standardized reach paths for controls vs. patients—(A): patient RB, (B): patient SS, and (C): patient AP—when grasping objects at midline with both the right and left hands in Experiment 2**.

**Figure 5:**
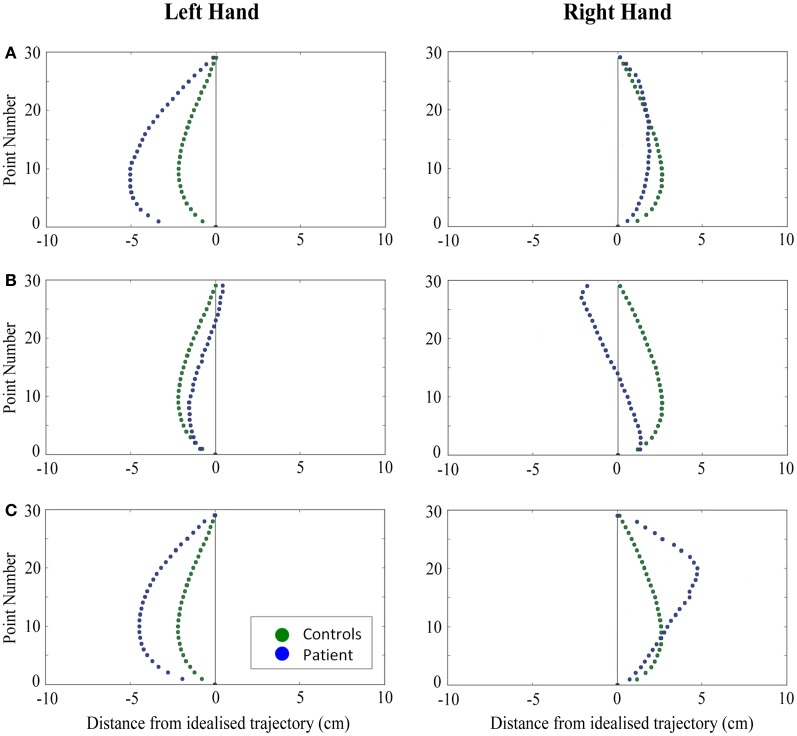
**A comparison of standardized reach paths for controls vs. patients—(A): patient RB, (B): patient SS, and (C): patient AP—when grasping ipsilaterally presented objects with both the right and left hands in Experiment 2**.

**Figure 6:**
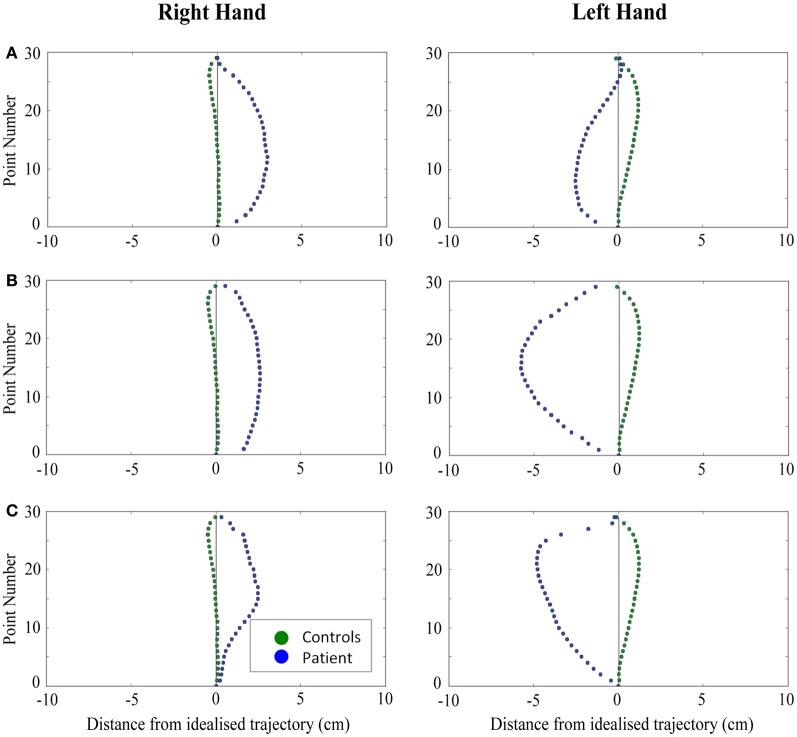
**A comparison of standardized reach paths for controls vs. patients—(A): patient RB, (B): patient SS, and (C): patient AP—when grasping contralaterally presented objects with both the right and left hands in Experiment 2**.

**Figure 7:**
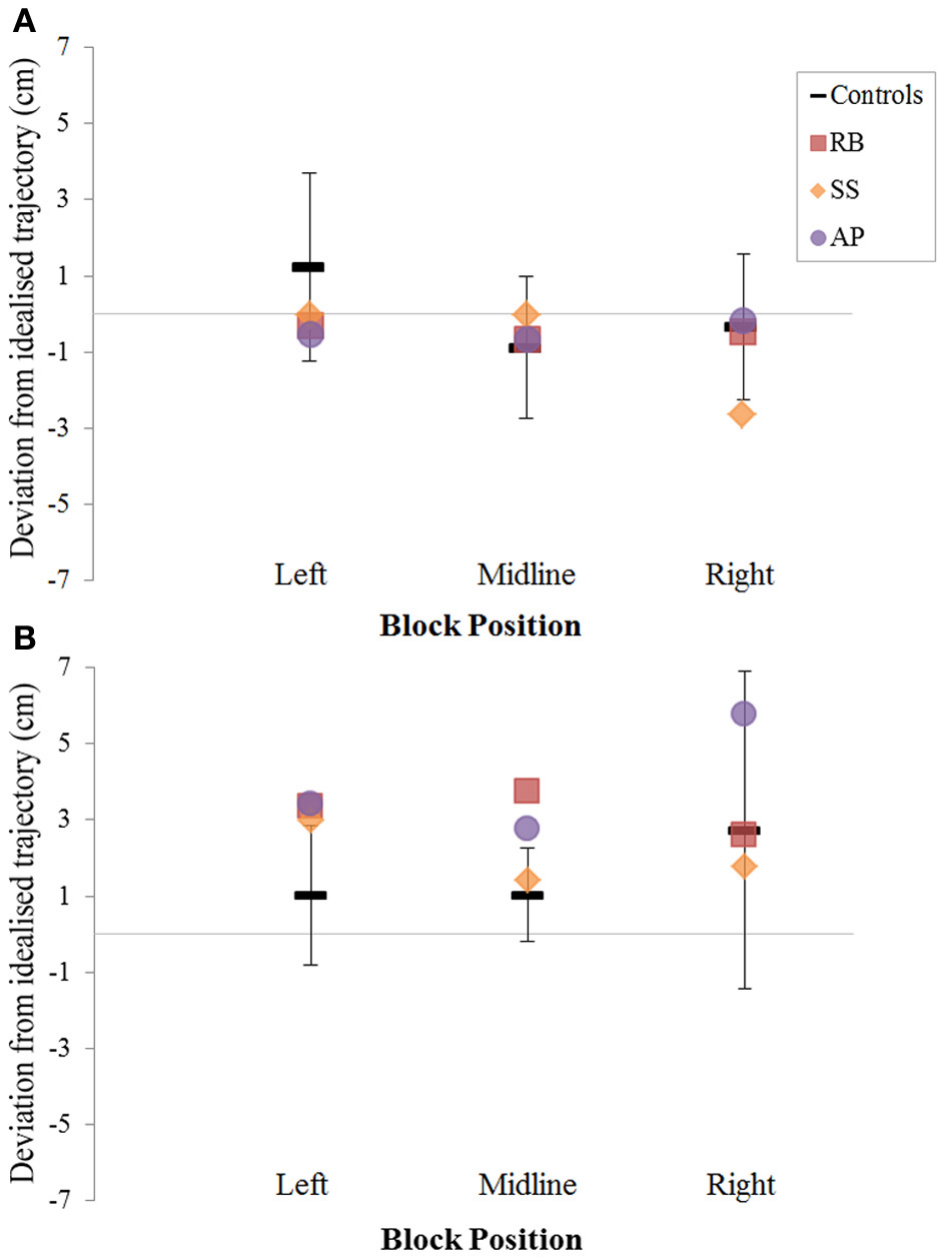
**The (A): leftward and (B): rightward reach path deviations in Experiment 2 for reaches made with the right hand**.

**Figure 8:**
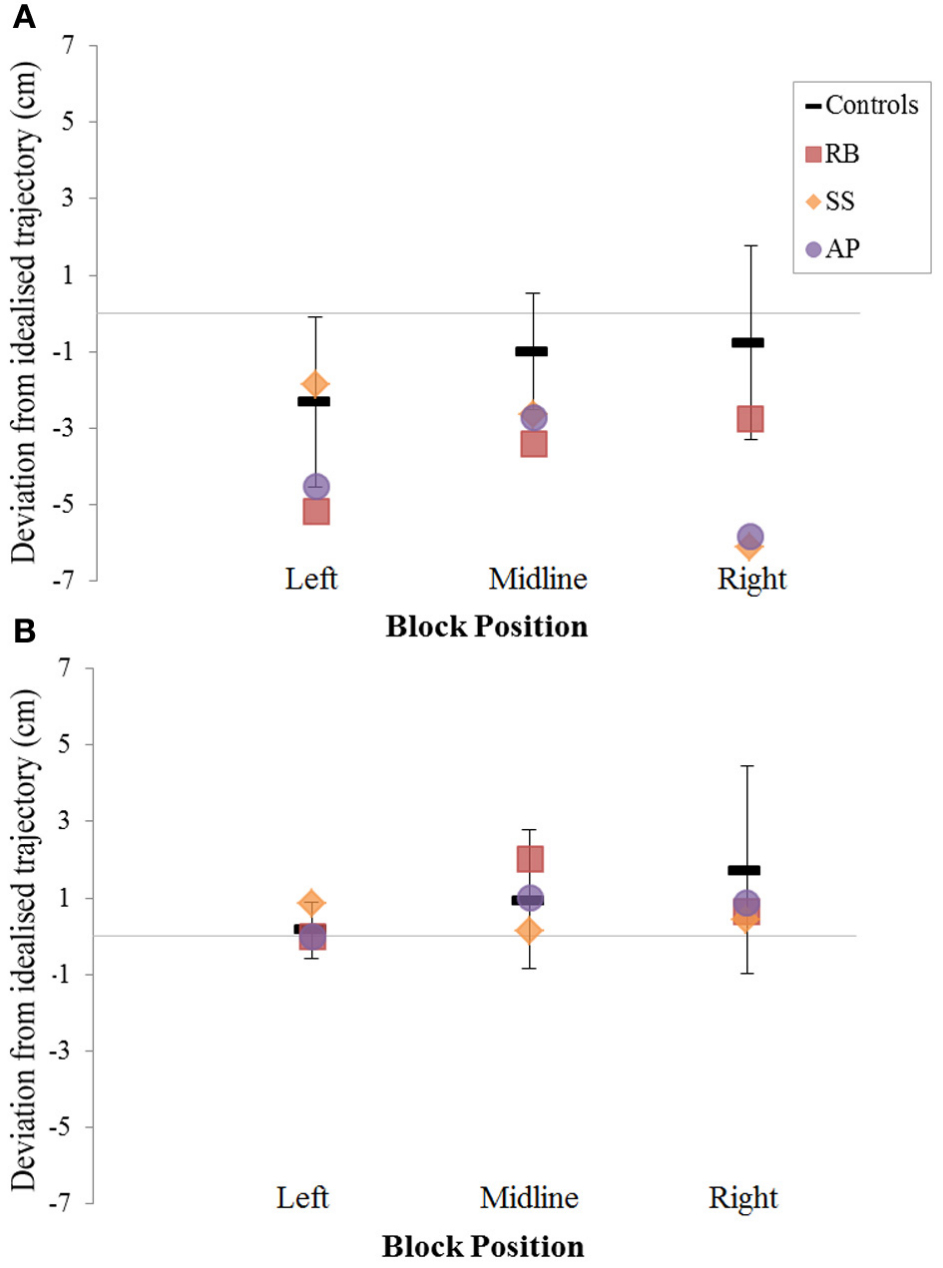
**The (A): leftward and (B): rightward reach path deviations in Experiment 2 for reaches made with the left hand**.

When reaching for objects at midline, controls showed slightly curved trajectories in the direction of the hand being used (Figure 4). However, these trajectories did not deviate significantly from the idealized straight-line path between initial and final wrist position (Figures 7, 8). During ipsilateral reaching, in which peripherally-presented objects were positioned on the same side of the body to that of the hand being used, controls exhibited a very similar pattern of behavior to that seen at midline: reach paths showed a tendency to curve away from the idealized straight-line path in the direction of the hand being used (Figure 5). This bias was significant for the left hand (Figure 8A), but not for the right (Figure 7B). During contralateral reaching, in which peripherally-presented objects were positioned on the opposite side of the body to that of the hand being used, controls once again showed trajectories that did not deviate significantly from an idealized straight-line path to the target (Figure 6). With respect to magnetic misreaching, only three of the 360 reaches made by controls (<1%) passed through the fixation point ROI. Closer inspection of these three reaches revealed that wrist height was maintained high above the surface of the table and there was no decrease in wrist velocity as the hand passed through the ROI. In other words, these rare deviations toward the fixation point simply represented a highly-curved trajectory to the peripheral target, rather than a reach directed specifically toward the fixation point.

#### Patients

***Patient RB.*** RB showed reach paths for objects at midline (Figure 4A) that deviated significantly more from the idealized straight-line path than those of controls with both hands (Figures 7B, 8A). Specifically, her trajectories were biased toward the side of the hand being used. For ipsilateral reaches (Figure 5A), RB's right-hand paths were very similar to controls (Figure 7), but she showed a significant deviation from the reach-path made by controls with her left hand in the direction of the hand being used (Figure 8A). For contralateral reaches (Figure 6A), RB showed a tendency to reach toward the point of fixation with both hands, though this deviation was only significantly different from controls with her right hand (Figure 7B). Considering only grasps to peripheral targets, three (5%) of RB's reach-paths passed through the fixation point ROI. A kinematic analysis of these three reaches revealed that as her hand passed through the ROI, her wrist was held high above the surface of the table and there was no decrease in wrist velocity. Therefore, like controls, the deviation toward the fixation point simply represented a highly-curved trajectory to the peripheral target, rather than a reach directed specifically to the fixation point.

***Patient MTB.*** MTB was unable to complete this task. While she had no trouble grasping objects placed at her midline, she found it impossible to execute grasps to objects in her periphery. When prompted to reach to peripheral targets, she would sit for a long time without moving, staring intently at the fixation point. When again prompted to reach to the target, she would sometimes reply that it was “too difficult.” Often she would move her hand two or three centimeters from the start button, keeping her fingers together, and place it back down on the table. MTB maintained that she could see the objects, but found it very difficult to initiate or complete a movement toward them. This experiment was attempted a second time with MTB, this time placing the peripheral objects 6 cm from the fixation point rather than 12 cm. This change made no difference to MTB's behavior: she reported that it was now easier to see the objects, but she was still unable to attempt grasps toward them.

An inability to initiate motor actions can be indicative of the presence of various neurological disorders, such as ideomotor apraxia, ideational apraxia, or akinesia. However, these explanations seem unlikely given MTB's intact ability to execute grasps to objects at midline in Experiment 1, as well as her ability to pantomime object use—observed during administration of the Edinburgh Handedness Inventory. The most reasonable explanation, therefore, is a combination of visual field defects and the presence of optic ataxia. As previously mentioned, MTB suffers from a generalized loss of sensitivity in both her left and right visual fields. As a result, MTB may have had trouble seeing the peripheral target objects clearly, despite her claims to the contrary. However, it is very possible that the visuomotor impairments seen in Experiment 1 also contributed to MTB's inability to perform this task.

***Patient SS.*** At midline (Figure 4B), SS showed reach trajectories that were much more comparable to controls than either RB or AP. However, the reach-path for his left hand did show a significant leftward deviation from that of controls (Figure 8A). For ipsilateral reaches (Figure 5B), SS showed a significant departure from controls with his right hand, reaching closer to the point of fixation (Figure 7A). He showed a similar trend toward the fixation point with his left hand, but the effect did not reach significance (Figure 8B). This fixation point bias was even more apparent for contralateral reaches (Figure 6B), where SS's reach paths showed a significant departure from controls toward the fixation point with both his right and left hands (Figures 7B, 8A). Considering only grasps to peripheral targets, nine (15%) of SS's reach-paths passed through the fixation point ROI. Looking at the kinematics of these atypical trajectories, we see that in five of SS's aberrant reaches, wrist height and velocity decreased as the hand entered the ROI, indicating a significant deviation from a normal peripheral grasp trajectory. In other words, SS demonstrated reaches that were drawn toward the fixation point to such an extent that the kinematics of his trajectory resembled those of a reach made to a central target. In fact, in the majority of these reaches, SS actually touched his fingers to the surface of the table at the fixation point, before “correcting” his movement and accelerating toward the peripheral target.

***Patient AP.*** AP's reach paths deviated from controls with both hands for objects located at her midline (Figure 4C). As with the other patients, her reaching bias was in the direction of the hand being used (Figures 7B, 8A). For ipsilateral reaches (Figure 5C), AP showed a trend toward reaching away from the point of fixation compared to controls. However, this effect was only significant for reaches made with her dominant left hand (Figure 8A). For contralateral reaches (Figure 6C), AP showed the same pattern as SS—reaching closer to the point of fixation than controls with both hands (Figures 7B, 8A). In order to determine whether these results were purely a consequence of AP's handedness, the path comparisons were repeated, comparing AP's path deviations for her left hand to controls' right hands, and vice versa. Instead of left and right hands reaching to left, midline, or right targets; conditions were now dominant and non-dominant hands reaching to ipsilateral, midline, or contralateral targets. Following this analysis, the results of AP's reaches to midline and contralateral targets did not change—her reaches at midline were biased toward the effector hand, and her contralateral reaches were biased toward the fixation point. However, she no longer showed a path bias with her left hand for ipsilateral targets, but instead showed a bias with her right hand for ipsilateral targets. As before, this reach bias was away from the point of fixation. This change in result was driven by the much higher variation in reach paths made by controls for the ipsilateral condition with their dominant, right hands compared to their non-dominant, left hands. Considering only grasps to peripheral targets, eight (13%) of AP's reach-paths passed through the fixation point ROI. In a similar manner to SS, the kinematics of AP's reaches through the ROI showed that in four of these eight reaches, AP's wrist height and velocity decreased as her hand entered the ROI. Additionally, her fingers occasionally touched the table during these reaches, as if she were executing a grasp to the fixation point itself rather than the peripheral target.

## Discussion

The goal of this investigation was to identify and quantify the nature of visuomotor deficits in a small group of individuals with PCA. Prior to testing, only one of the individuals in the PCA group, MTB, self-reported visuomotor issues, which manifested as trouble accurately picking up objects in front of her. Instead, perceptual issues, such as “fuzzy vision,” difficulty reading, and problems with object and face recognition, were presented as being the most pervasive and intrusive issues in the patients' lives. Preliminary testing confirmed that RB, MTB, and SS all have great difficulty completing tasks that require proper perceptual and visuospatial functioning, including the recognition and matching of geometric shapes, objects, and faces. In contrast, AP's basic face and object recognition is relatively intact, although she struggles with more complex tests such as the Benton Visual Form Discrimination task. Despite the minimal self-reporting of visuomotor deficits, Experiments 1 and 2 revealed that all four PCA patients demonstrate varying degrees of visuomotor malfunction. Kinematic abnormalities, such as a lack of grip scaling (in the case of RB) and a lowered peak wrist velocity (in the case of MTB) were seen when patients picked up simple, rectangular objects, presented at their midline. Visuomotor errors were compounded when patients were asked to reach for objects in their visual peripheries or when visual feedback of their reach was removed.

### Reach kinematics

The presence of grasping dysfunction, which included impaired grip scaling and protracted movement durations, for reaches to central targets in all four PCA individuals was unexpected. Examples of patients with optic ataxia demonstrating movement deficits under foveal guidance have been previously reported (Perenin and Vighetto, 1988; Jeannerod et al., 1994; Binkofski et al., 1998), however, most patients with optic ataxia demonstrate preserved accuracy for movements toward objects located in central vision (Rossetti et al., 2003). A number of possibilities exist to explain this finding. First, it is possible that the cortical damage in the PCA patients is more extensive, or at least more cortically widespread, than is typically seen in previously reported cases of optic ataxia. This more distributed damage may be affecting a larger number of neural control systems that might ordinarily be able to compensate for one another in cases of discrete lesions. In favor of this explanation is the presence of diffuse atrophy affecting extensive posterior cortical regions in anatomical scans of the PCA group. Secondly, it may be the case that our very precise kinematic recordings picked up minor reaching and grasping abnormalities that would not otherwise have been noticed. In favor of this explanation is the observation that all four individuals with PCA were able to successfully execute relatively smooth reaches and stable grasps to different-sized objects at midline under visual guidance. Instead, visuomotor abnormalities presented themselves as impaired grip scaling and slower movements; abnormalities that were only apparent following careful analysis of kinematic recordings.

Prior to this experiment, we hypothesized that PCA patients with predominant dorsal steam deficits might experience an improvement in their grasping ability following visual occlusion. This hypothesis came from reports of optic ataxic patient IG, who was unable to properly scale her grasps when reaching to peripheral targets under visual guidance, but showed appropriate grip scaling during delayed real grasping and delayed pantomime grasping (Milner et al., 2001). However, the observation of extensive perceptual issues and diffuse atrophy in areas of cortex making up the ventral stream indicated that our patients were unlikely to mirror the behavior shown by patient IG. Indeed, Experiment 1 revealed that none of the four patients in the current study demonstrate a comparable improvement in reaching and grasping abilities when visual feedback is removed. In fact, MTB, SS, and AP, who are able to scale their grasps to the size of objects under visual guidance, show much poorer performance on the reach-to-grasp task following a delay. These results suggest that all four PCA patients in the current study suffer from damage to temporal cortical areas that are ordinarily able to provide visuomotor systems with lasting perceptual representations of the environment. This conclusion is especially telling for AP, who demonstrates the least severe perceptual problems.

### Reach trajectories

Experiment 2 revealed that RB, SS, and AP all produce highly curved reach trajectories, both at midline and in the periphery. This behavior presents a stark contrast to the relatively straight reach paths produced by controls. It is unclear exactly why the patients produce these excessively curved reach paths, but it is clear that their visuomotor systems are not programming reach trajectories in the most efficient manner. In Experiment 1, we saw that three of the individuals in the PCA group show slower, protracted reaching movements. Rossetti et al. (2003) suggest that the slowing down of movements may be an attempt to compensate for damaged on-line control centers in the parietal cortex by engaging slower visual feedback loops that can refine the programming of movements based on incoming visual information. It is possible that the curved reach trajectories in Experiment 2 are related to these results in that the patients prefer an unobstructed view of the target object throughout the reach in order to monitor their grasping movements and guide their fingers to stable grasp sites on the objects. Highly curved trajectories, especially for reaches at midline, would maximize such observation by removing the effector hand from the line of sight between the eyes and the target object.

At times, SS and AP both demonstrate reach kinematics that resemble reaches directed toward the fixation point itself, rather than toward the peripheral target. This behavior, termed magnetic misreaching, has been previously documented in patients with parietal lobe dysfunction (e.g., Carey et al., 1997; Jackson et al., 2005). Jackson et al. (2005) have suggested that magnetic misreaching occurs when the visuomotor systems underlying eye and limb movements are not properly uncoupled in order to allow for each system to perform simultaneous but independent actions. Eye and hand movements are highly coordinated processes (Fisk and Goodale, 1985), as demonstrated by the tight temporal and spatial coupling seen between gaze fixation and grasp site locations (e.g., Desanghere and Marotta, 2011). The robust cooperation between eye movements and hand guidance underlies our ability to quickly and accurately interact with objects around us. Of course, the dissociation between eye and hand movements is not only possible, but also necessary, for efficient and versatile object-interaction. Independent control of these two processes underlies our ability to execute a reach without direct visual guidance, or to shift our gaze away from an ongoing manual task.

It has been suggested that the uncoupling of gaze and prehension relies on cortical systems that send inhibitory signals to midbrain structures (Milner et al., 2003), such as the superior colliculus, and recent studies have identified the parietal-occipital junction as a potential source of this inhibition (Clavagnier et al., 2007). Milner et al. (2003) suggest that magnetic misreaching may represent a “primitive” form of reaching, in which inhibition of midbrain structures is lost due to cortical damage. However, recent neurophysiological studies in both primates and humans have identified the presence of strong reach-related signals in certain neurons in the superior colliculus, including during reaches made to peripheral targets (Reyes-Puerta et al., 2010; Song et al., 2011; Linzenbold and Himmelbach, 2012). Based on this evidence, Linzenbold and Himmelbach (2012) suggest that the superior colliculus may play a much larger role in hand-eye coordination than previously thought, though it remains unclear to what extent the superior colliculus has the ability to modulate cortical motor commands. In the current study, SS and AP showed strong evidence of magnetic misreaching, and all four patients had difficulty suppressing the urge to look at the object to which they are directing their reach. This behavior suggests that these individuals have difficulty inhibiting the powerful coupling of eye and hand movements occurring at either the cortical or subcortical level.

An important potential limitation of this experiment is the presence of visual field defects in three of the four individuals with PCA—RB, MTB, and SS. As previously discussed, MTB's inability to perform the peripheral task in Experiment 2 may be in large part due to her hemianopia. If she was unable to accurately locate the target objects in her peripheral vision, then she may have been unwilling to “blindly” attempt a reach. However, MTB's hemianopia is limited to her left visual field, so she should have been able to at least attempt reaches to right-side targets if visual field deficits were indeed the root-cause of her problems. RB and SS both suffer from quadrantanopia, primarily affecting their upper fields of vision, which may have had a significant effect on their ability to accurately perform the peripheral grasping task. Interestingly, however, AP showed similar “magnetic misreaching” behavior to that of SS, yet visual field testing showed her peripheral vision to be unaffected by field defects.

### Ventral and dorsal subtypes of PCA?

None of the patients in the current study demonstrate symptomology indicative of a purely ventral or a purely dorsal subtype of PCA. However, despite the highly mixed symptomology presented by our patients at the time of testing, their histories tell a slightly different story. RB initially presented to her neurologists with only perceptual complaints, while MTB suffered from primarily visuomotor problems. Given the length of time these individuals have been experiencing symptoms, it is perhaps not surprising that they all now exhibit more of a mixed disorder. The current study shows no evidence for the presence of purely ventral or dorsal subtypes of PCA in a small group of patients, but suggests that individual patients may experience primarily ventral or dorsal symptoms early on in the disease. As such, a ventral/dorsal subclassification of PCA may be useful in identifying the primary site of atrophy early on in PCA, but seems to become less valuable as the disease inevitably progresses to a state of more diffuse cell loss.

## Concluding remarks

It is our belief that PCA is a more common affliction than is generally reported. One of the goals of the current research was to improve the general understanding of PCA symptomology, thereby helping clinicians to better identify and differentiate PCA from other medical conditions. For most of our PCA patients, it was a long trial-and-error process working with their family physicians and optometrists before they were properly diagnosed by a neurologist. We hope that this research will help expedite diagnosis, which would allow patients with PCA to receive the correct medical treatments earlier in the disease. Generating a better understanding of how PCA symptoms manifest themselves may help physical and occupational therapists to better tailor their care programs when working with PCA patients, and may facilitate the creation of coping strategies for patients and caregivers.

### Conflict of interest statement

The authors declare that the research was conducted in the absence of any commercial or financial relationships that could be construed as a potential conflict of interest.
